# Supplementary material for: Convolutional neural networks explain tuning properties of anterior, but not middle, face-processing areas in macaque inferotemporal cortex
Source: Commun Biol. 2020 May 8;3:221. doi: 10.1038/s42003-020-0945-x (PMC7210114; doi:10.1038/s42003-020-0945-x)
Supplement: Supplementary file 4 — Description of Additional Supplementary Files [file 42003_2020_945_MOESM4_ESM.pdf]

Description of Additional Supplementary Files

File Name: **Supplementary Data 1**
Description: an Excel file that contains all the numerical values in the main figures from our simulations.
